# Acoustic-Signal-Based Damage Detection of Wind Turbine Blades—A Review

**DOI:** 10.3390/s23114987

**Published:** 2023-05-23

**Authors:** Shaohu Ding, Chenchen Yang, Sen Zhang

**Affiliations:** 1 College of Mechatronic Engineering, North Minzu University, Yinchuan 750021, China; 2 College of Electrical and Information Engineering, North Minzu University, Yinchuan 750021, China

**Keywords:** acoustic signal, non-destructive testing, wind turbine blades, machine learning

## Abstract

Monitoring and maintaining the health of wind turbine blades has long been one of the challenges facing the global wind energy industry. Detecting damage to a wind turbine blade is important for planning blade repair, avoiding aggravated blade damage, and extending the sustainability of blade operation. This paper firstly introduces the existing wind turbine blade detection methods and reviews the research progress and trends of monitoring of wind turbine composite blades based on acoustic signals. Compared with other blade damage detection technologies, acoustic emission (AE) signal detection technology has the advantage of time lead. It presents the potential to detect leaf damage by detecting the presence of cracks and growth failures and can also be used to determine the location of leaf damage sources. The detection technology based on the blade aerodynamic noise signal has the potential of blade damage detection, as well as the advantages of convenient sensor installation and real-time and remote signal acquisition. Therefore, this paper focuses on the review and analysis of wind power blade structural integrity detection and damage source location technology based on acoustic signals, as well as the automatic detection and classification method of wind power blade failure mechanisms combined with machine learning algorithm. In addition to providing a reference for understanding wind power health detection methods based on AE signals and aerodynamic noise signals, this paper also points out the development trend and prospects of blade damage detection technology. It has important reference value for the practical application of non-destructive, remote, and real-time monitoring of wind power blades.

## 1. Introduction

**Figure 1:**
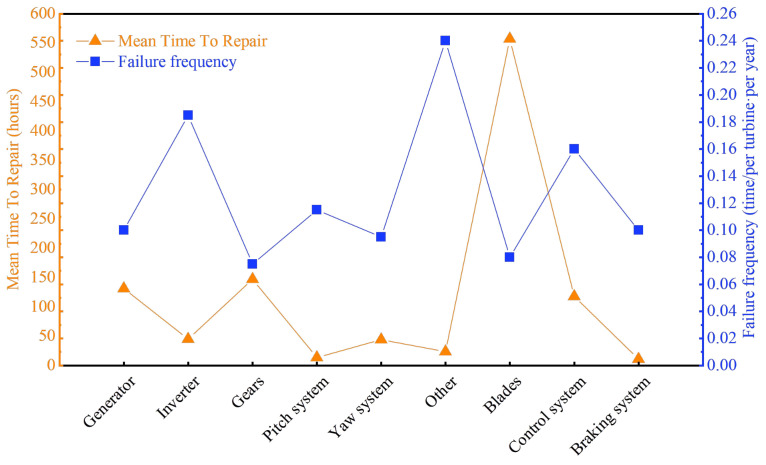
Frequency of failures and repair time of wind turbine components based on data retrieved from [5].

In recent years, global climate change has become a key issue for all of mankind. With the continued warming trend, environmental protection actions are imminent. Therefore, the development of clean energy is an important solution to cope with environmental changes. Wind power is favored by countries in all regions of the world because of its zero-carbon-emission advantage. According to the Global Wind Energy Council’s (GWEC) 2022 Global Wind Energy Report [1], wind power capacity is expected to reach 557 GW by 2026. With the rapid growth of installed wind power capacity, the wind power industry is facing various challenges. Wind turbine operation fault monitoring and maintenance technology have become key issues restricting the development of the wind power industry. Ribrant and Bertling [2] investigated the critical failures of wind turbines in Swedish wind farms and analyzed the percentage of failures and downtime of different components for maintenance. The China Renewable Energy Professional Committee investigated the operational quality of wind power and the frequency of failures, as well as the corresponding repair time (blue and orange dashed lines, respectively, in Figure 1), of wind turbine components derived from the survey data. As shown in Figure 1, blades are the components responsible for the highest frequency of wind turbine failures and associated with the longest repair time. Therefore, the development of wind turbine blade health detection technology has become an urgent need for the wind energy industry. In practice, wind turbines can only generate maximum power within a specific wind speed distribution range [3]. However, the actual wind speed will change, requiring timely and accurate regulation of wind turbines. To solve the problem of not always running in sync with factory mode due to quantization, time delays, and ubiquitous data dropouts, Cheng et al. [4] designed a Dissipativity-based finite-time asynchronous output feedback control for a wind turbine system via a hidden Markov model. A simulation example of a wind turbine system is used to verify the correctness and applicability of the controller.

**Figure 2:**
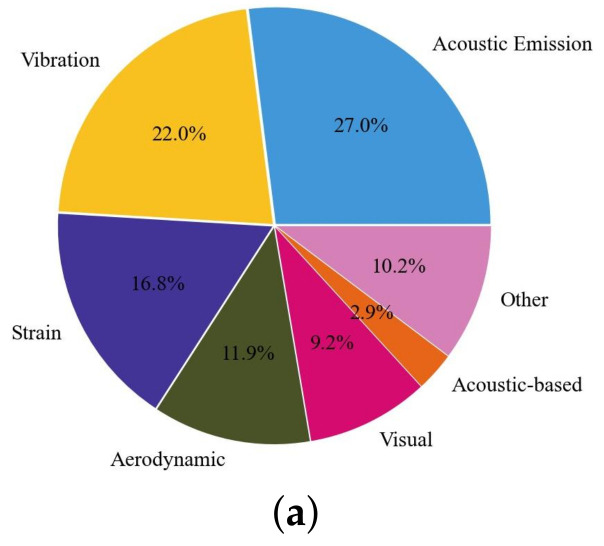

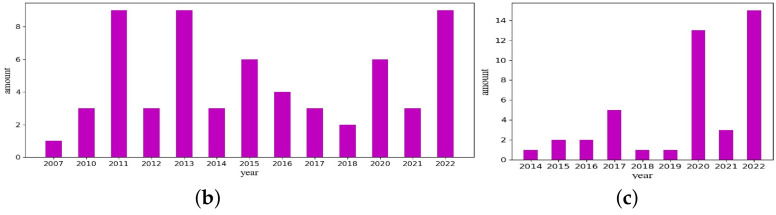
(**a**) Prevalence of wind turbine damage detection methods in the literature. The number of studies published in the literature based on (**b**) AE detection and (**c**) aerodynamic noise detection.

At present, the methods used for wind turbine blade health monitoring include acoustic emission, vibration detection, strain detection, aerodynamics, machine vision, thermal and sound-based features, etc. In this review, we analyze different damage detection techniques for wind turbine blades through a search of the Web of Science core collection. A search using the terms “wind turbine blade” AND “damage” yielded a total of 1410 articles. We performed an econometric analysis of the statistical data available to date using Python, as shown in Figure 2, indicating the relative prevalence of major blade damage detection techniques in the literature. This paper focuses on the application of AE damage detection and aerodynamic noise damage detection technology in wind turbine blades. Figure 2b is blade aerodynamic noise and Figure 2c show the research trends of non-destructive testing (NDT) techniques in recent years.

### 1.1. Vibration Detection

When the structure of a wind turbine blade is damaged, its structural vibration response reflects corresponding damage information. Ghoshal et al. [6] achieved damage detection before a catastrophic failure of the blade by analyzing the blade vibration response. Abouhnik and Albarbar [7] provided an overview of wind power vibration sources. A new method using the empirical decomposition feature intensity level (EDFIL) was proposed for analysis of vibration signals and identification of crack damage. Chen et al. [8] studied the sensitivity of vibration modes of blades of different sizes to damage recognition and found that when blade size increased significantly, vibration modes were relatively sensitive to damage recognition. In order to optimize the disadvantage that the natural vibration modes are not sensitive to small blade damage, Fremmelev et al. [9] designed an active monitoring system consisting of an electric shaker and a distributed accelerometer. Fatigue experiments were also carried out for multiple artificial damages introduced into blades successively. In addition, machine learning algorithms have been introduced for the analysis of vibration signals. Dervilis et al. [10] combined an artificial neural network (ANN) algorithm with vibration response for detection of the structural integrity of wind turbine blades. Khazasee et al. [11] used a convolutional neural network (CNN) model to classify vibration data with respect to blade health and fault states. Ogaili et al. [12] integrated machine learning and finite element analysis methods for wind turbine blade damage detection. However, signal-based damage detection is associated with a certain time lag in vibration, and the damage detection function can be achieved only when the damage exceeds the rated threshold. The number of vibration-signal-based damage detection sensors, the signal-to-noise ratio, wind speed, and blade speed are all factors that limit vibration-signal-based NDT for wind turbine blade fault detection.

### 1.2. Strain Detection

Fiber Bragg grating (FBG) sensors are commonly used for strain detection. Once the sensor is subjected to stress or temperature changes, the grating pitch changes, which affects the wavelength of the reflected light to meet the detection requirements. Li et al. [13] used FBG sensors to study material strain data from layered fabricated structures and to obtain information on the performance and structural integrity of wind turbine blades in harsh environments. Krämer et al. [14] used an FBG network embedded in the composite structure of a wind turbine blade to measure blade strain data. However, FBG sensors also have their own limitations in wind turbine blade damage detection, such as uncertainty of the damage to the material structure caused by the embedded installation method, the impracticality of a large blade installation, and the difficulty associated with direct damage detection.

Digital image correlation (DIC) is another strain detection technique used for materials that calculates the deformation information of two digital images before and after damage, representing the displacement change in the region. In recent years, some newer DIC techniques have been proposed [15,16,17], such as a DIC technique using the AC-SURF matching algorithm for damage detection of rotating blades. However, DIC detection results are easily disturbed by occlusions. Furthermore, high-accuracy resolution acquisition equipment and high detection costs are required to improve the accuracy of detection results. On the other hand, the DIC technique makes it difficult to detect damage to the internal material of the blade.

### 1.3. Aerodynamic Detection

Damage causes changes in the aerodynamic parameters of blades relative to structurally healthy blades. Wu et al. [18] investigated the unsteady aerodynamic load response and strain distribution on blades. Fatigue damage was also predicted using unsteady aerodynamic loads, Goodman diagrams, and Miner linear superposition. Wang et al. [19] combined the finite-element method for kinetic analysis with the use of the vibration differential curvature for damage diagnosis in order to perform blade damage detection. Zhang et al. [20] used the estimated values of blade loads, stress analysis, and fatigue crack evolution to analyze and evaluate blade damage. However, blade dynamics analysis is a very complex process. Wind shear, tower–blade interaction, aeroelasticity, and rotational effects may affect damage evaluation results. Furthermore, the blades are easily damaged by the erosion of large solid particles of air, which directly affects the power output. Lee et al. [21] analyzed the transient three-dimensional computational fluid dynamics of blade tip suction surface erosion shape and divided it into four grades. However, an accurate particle flow representation model is crucial to the analysis results. Therefore, conducting particle hydrodynamics analysis should be combined with specific erosion models.

### 1.4. Visual Inspection

Infrared thermography is a method used to monitor the changes in heat conduction inside a structure, generating thermal images for the detection of object damage. Dattoma et al. [22] examined the material thermal response of wind turbine blades inflicted with different artificial defects by thermography to verify the reliability of the thermographic method. Doroshtnasir et al. [23] proposed long-range detection of wind turbine blade surface defects by minimizing interference. Zhang et al. [24] used the equivalent source method (ESM) to establish a three-dimensional model based on heat transfer theory to detect wind turbine blade wing beam caps. In addition to the thermal characteristics caused by deposits on the blade surface, other inhomogeneous materials can affect the thermal imaging results. Especially when used for composite wind blade interlayer damage detection, imaging results may be disturbed by material anisotropy and inhomogeneity, resulting in significant noise.

Visual inspection is a common engineering damage detection method used for wind turbine blades. Xiao et al. [25] used an unmanned aircraft to collect images of wind turbine blades, which they combined with an Alexnet classifier to automatically diagnose blade surface damage. Gunturi et al. [26] used super-resolution convolutional neural networks to convert blurred images into high-resolution images in combination with the Yolo-v3 neural network for wind turbine blade damage pattern recognition. In addition, Mao et al. [27] proposed a cascaded depth network superior to models such as YOLO-v3 for the automatic detection of multiple types of surface damage to wind turbine blades. Despite the rich variety of visual inspection methods and the intuitive presentation of results, irrelevant obstacle occlusions remain an important limitation of visual detection.

### 1.5. Other Detection Methods

Other techniques have also been used to study blade damage, in addition to those listed above. Ultrasonic inspection is a method whereby changes in physical parameters such as propagation, reflection, and attenuation of ultrasonic waves in materials are used to detect damage. Bird et al. [28] reviewed the development of ultrasonic techniques and described a technique for early crack detection in wind turbine blades. However, ultrasonic detection of damage generally requires contact with the surface of the object of interest. Therefore, a portable long-distance ultrasonic propagation imaging (LUPI) system was proposed and used for blade damage identification in operating wind turbines [29]. However, the research of ultrasonic damage detection is limited by the mode of dispersion and number of wireless for a long time, which becomes a big challenge to the use of ultrasonic detection. Recently, a suitable error and uncertainty estimation method has been proposed, as well as the reliability of the defined measurement parameters for Lamb wave signal processing methods for dispersion evaluation, validation, and help to determine damage [30]. To further improve the detection accuracy, Duernberger et al. [31] proposed a damage detection method using a multiaperture (MA) acoustic beamforming technique enabling a significantly higher detection speed compared to conventional linear beams. Therefore, the proposed technique is suitable for real-time monitoring and warning. Ultrasonic NDT techniques are still subject to signal processing technology limitations, such as high signal-to-noise ratio requirements for long-range detection, interference from other signals in wind turbine blade multilayer composites, and high-precision detection hardware requirements.

Various types of ray inspection have also been applied to blade damage detection. For example, X-ray technology has been used for detection of blade delamination damage, bending or impact fatigue damage, leading edge erosion, and rain erosion [32,33,34,35,36,37]. Recently, terahertz radiation (T-ray) has received increasing attention for blade inspection. Currently, T-ray inspection techniques are most often used for composite wind turbine blade impact damage and wind turbine blade trailing edge cracking damage detection [38,39,40,41]. However, composites consisting of carbon fibers can limit the propagation of T-rays. Therefore, terahertz inspection techniques are not suitable for detecting defects in carbon fiber composite wind turbine blades. On the other hand, microwave scanning technology based on open waveguide sensors is also used to detect blade damage [42]. However, high-frequency microwaves cannot penetrate the blade structure. Al-Yasiri et al. [43] used UAV to emit electromagnetic waves below the microwave frequency to detect blade crack damage. Eddy current thermography with pulsed microwaves can also be used for blade damage detection. This method has the advantages of a fast detection speed and a relatively wide defect range for identification of composite blade impact damage, surface cracks, and delamination damage [44,45,46].

Recently, shearography has been used for wind turbine blade damage detection due to its applicability to composites and its remarkable sensitivity [47]. Maierhofer et al. [48] explored the effect of delamination defects on different diameters, depths, and isotropic fibers using flash and locked thermography. Li et al. [49] proposed a blade damage detection technique using phase shift and dynamic thermal loading. Recently, a robot that can carry a shear imaging unit was studied and deployed for the detection of blade damage [50].

Acoustic-signal-based wind turbine blade detection technology has been widely studied and applied owing to its advantages such as high detection accuracy, remote implementation capabilty, and easy sensor installation. Blade detection technology based on acoustic signals can be divided into AE signal and aerodynamic noise signal detection. AE signals differ significantly from aerodynamic noise signals in terms of their generation principle and frequency range. An AE signal is an ultra-high-frequency stress wave release pulse signal caused by material stress changes and crack excitation, with a frequency that usually exceeds 20 kHz [51]. The aerodynamic noise signal of wind turbine blades is generated by their interaction with air blowing towards the blades. The frequency is mainly distributed in the audible range of 200 Hz to 20 kHz [52]. Techniques for blade structural integrity detection based on AE and aerodynamic noise signals are described in further detail in the following sections.

According to the existing literature, wind turbine blade structural integrity detection and damage location detection is an interesting research field. Unlike previous research, this paper first reviews the research results of acoustic emission signals and aerodynamic noise signals on wind turbine blade damage in recent years. Then, the application of these two signals in blade damage detection and damage source location is analyzed. Finally, the development trend and prospect of wind power blade damage detection technology are discussed, which will provide reference for understanding blade damage types and blade damage detection technology. The main contributions are summarized as follows:

1. The traditional analysis methods of AE signals are reviewed, and the monitoring of leaf damage evolution and damage location by AE signals are analyzed.
2. This paper introduces two types of noise generated by wind turbines. The types of aerodynamic noise and the possible factors affecting the variation in aerodynamic noise characteristics are discussed. In particular, the methods and applications of blade damage monitoring based on aerodynamic noise are reviewed and analyzed.
3. A machine learning algorithm based on acoustic signals (AE, aerodynamic noise) of blades is analyzed to complete the task of automatic classification of blade damage.

## 2. Detection and Source Localization of Wind Turbine Blade Damage Based on AE Signals

### 2.1. Detection Method Based on AE Signals

The goal of the AE NDT technique is to discover AE sources through AE sensor monitoring and obtain as much information as possible using signal processing techniques. At the same time, certain characteristic information of the AE signal is linked to the changes in the material in order to achieve the monitoring of the structural health of the material. In the real environment, AE signals are complicated by their own characteristics, such as sensor characteristics, material properties, propagation paths, etc. [53]. According to the characteristics of the received AE signals, the analysis methods used to obtain AE source-related information are usually divided into two categories [54,55]. The first category is analysis methods based on analysis of time- and frequency-domain waveforms (including spectrum and correlation functions) using AE signals; the second category is analysis methods based on characteristic parameters with simplified waveforms.

#### 2.1.1. Waveform Analysis

Based on AE waveform analysis, we can understand the process characteristics of the damage occurring in the material. For example, the difference between blade crack damage and edge damage was observed from the AE waveform time domain, and the edge damage waveform exhibited significant secondary shock [56]. Other characteristics of the AE signal have also received attention; for example, Van Damet et al. [57] investigated the different mode waves of pencil cores excited by in-plane and out-of-plane fractures. The authors found that stretching waves propagate faster than bending waves. De Souza Rios et al. [58] studied AE signal waveforms during the aging of three different composite thicknesses and found that the acoustic wave propagation velocity and attenuation coefficient were changed. Moreover, microscopic cracks, matrix macroscopic cracks, and fiber fracture damage formed in the material by adhesive porosity were analyzed in tensile and compression experiments. The results show that the frequency interval of the waveform varies for the same damage type due to different loading levels [59].

AE signals are very complex. Variations in wave velocity, attenuation, and damage frequency range due to different compositions of composite materials (material composition and material thickness), special geometry, loading methods, and sensor placement may be the reason for the difference in analysis results. Therefore, frequency-domain and time–frequency-domain signal processing methods can be used to analyze the propagation characteristics of AE signals under the influence of different materials or the characteristics of different failure mechanisms. The frequency-domain waveform analysis method can obtain the wave patterns of AE signals excited in a specific material and at a specific location. For example, Fotouhi et al. [60] investigated the frequency range of different excitation modes causing various forms of damage in composite materials. The results of Fourier transform (FT) analysis also proved that defects such as wind turbine blade matrix cracking and fiber fracture correspond to different frequency ranges. Although frequency domain analysis can be used to observe the frequency components contained in the signal to distinguish the damage type or the fault damage severity level, analysis of the wind turbine blade damage evolution process is limited by time sequence development. Unlike time domain analysis, the time–frequency-domain analysis method can be used to obtain signals containing frequency components and observe the relevant failure event time series [61,62,63,64]. As suggested by a study by Arumugam, waveform comparison and fast Fourier transform (FFT) can be used to label specific damage signals. Differences in matrix cracking, fiber debonding, and fracture were analyzed based on numerical differences in acoustic emission duration, amplitude, and energy-related parameters. Short-time Fourier transform (STFT) algorithms were obtained for the failure event time series [61].

Waveform analysis not only provides an understanding of the AE characteristics of the material damage process but can also aid in monitoring material failure. In order to establish a richer correlation with the damage process, other characteristic parameters must also be correlated. Classical characteristic parameters are obtained from waveform analysis using signal processing techniques in the time or frequency domain.

#### 2.1.2. Parameter Analysis

AE parametric analysis is a widely used method for investigation of AE signals. Most studies conducted to date have typically used AE parameters such as hit counts, amplitude, energy, duration, peak frequency, and other parameters to characterize AE failure mechanisms [65]. AE parameters include those obtained directly from AE sensors with suitable threshold settings or by selecting a suitable type of transducer (piezoelectric transducer), such as hit count, amplitude, and energy characteristic parameters. They also include characteristic parameters obtained using signal processing methods such as peak frequency.

**Figure 3:**
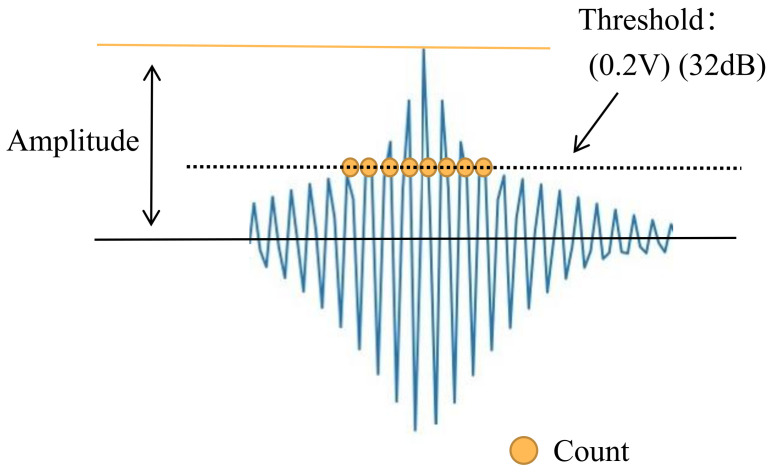
Count of AE signals.

Takaoka et al. [66] proposed a method to calculate the number of hits for fault determination. The intensity of the AE signal is measured at various wind speeds and compared to 32 dB as a threshold for distinguishing between normal and fault states. As shown in Figure 3, signals with amplitudes exceeding 32 dB are counted. Kim et al. [67] investigated the relationship between damage and AE signal amplitude distribution of carbon fiber reinforced polymer (CFRP) composites used for wind turbine blades by loading–unloading experiments. The experimental results showed that high-amplitude, medium-amplitude, and low-amplitude AE signals corresponded to three damage mechanisms, namely fiber fracture, debonding, and matrix cracking, respectively. Yajuan et al. [68] conducted tensile experiments on wind turbine blade composites containing type I delamination defects. The changes in characteristic parameters such as amplitude, hit count, and relative energy of AE signals were investigated, and the damage process and degree of damage to the composites were analyzed.

Wind turbine blade fault detection can be accomplished using simple acoustic emission characteristic parameters such as hit times. However, this method is highly susceptible to the interference of other factors in the environment and cannot provide a clear understanding of the differences between the failure modes. Therefore, characteristic parameters extracted based on the frequency domain or the time–frequency domain are usually used to analyze the failure events contained in AE signals.

### 2.2. AE Signal-Based Wind Turbine Blade Damage Detection and Localization

#### 2.2.1. Wind Turbine Blade Damage Detection

**Figure 4:**
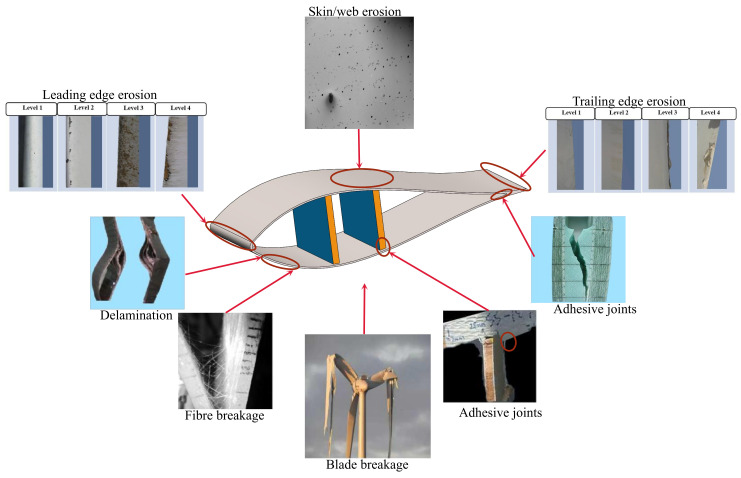
Types of wind turbine blade damage. Based on data retrieved from [81,82,83,84,85,86].

Wind turbine blades work for a long time in a field environment with complex conditions, and various harsh environmental factors, such as rain erosion, icing, lightning, and fatigue, can potentially damage blade materials. Typical manufacturing materials for wind turbine blades include glass-fiber-reinforced plastics and resins (GFRP), with a small proportion comprising wood composite materials [69,70,71,72] or other types of composites [73]. Although GFRP has the advantages of high strength, low mass, corrosion resistance, ease of fabrication, and low cost, weak interlaminar properties are a key factor affecting the lifetime of large wind turbine blades made with glass-fiber-reinforced composites [74,75]. Typical damage commonly observed in wind turbine blades is shown in Figure 4, for example, matrix cracking [76], delamination [77,78], fiber/matrix interface debonding [79], and fiber fracture [80].

Microcracks in the matrix are usually the first damage pattern exhibited by wind turbine blades. The cause may be long-term erosion damage to the blade caused by a harsh environment or potential damage caused by humans during manufacturing. Castorrini et al. [87] investigated the sources of blade damage under the influence of different environmental factors, such as high winds, sand, and rainfall, and determined that rain erosion is the main form of damage. Fraisse et al. [88] used experiments to simulate the damage process of rainwater erosion on glass fiber polymer laminates coated with gel layers. Each impact of the pellets causes a transient stress release in the blade laminate, which can produce cracks after multiple impacts. This finding may well help explain why cracks form in blades. It is interesting to note that the initial fatigue time is not easy to detect obvious damage, but can be regarded as damage accumulation latency. However, the initial fatigue time did not enable detection of significant damage but could be considered the damage accumulation latency period. Therefore, the FFT algorithm was used to study the wave frequency component of the almost invisible damage. The authors found that the wave frequencies changed significantly from before to after the impact damage [89]. With increased blade operating time, the blade’s latent phase damage accumulated into visible tiny cracks. These tiny cracks and their further development are the main cause of blade fatigue damage. Eventually, such damage develops into localized blade damage, matrix cracking, and even blade fracture. Niezrecki et al. [90] inserted tissue defects of defined geometry at specified locations in a 1 mm long blade and successfully detected sprouting cracks via AE cumulative energy release mapping. Li et al. [91] proposed a tracking algorithm to identify the first crack by sparse reconstruction using Lamb wave propagation characteristics in composite blades. The method was able to identify and trace transverse cracks with a length of 7 mm, achieving satisfactory accuracy of 1 mm × 1 mm in an experimental section of 150 mm × 80 mm. However, this method is only suitable for damage crack tracking in specified conditions and lacks universality of blade damage detection. Jee et al. [92] studied the fatigue damage characteristics of a blade with a length of 48 m. Analysis of the total impact energy and rise time revealed that the blade damage steadily increased. Therefore, the rise time can be relied upon to determine the generation and increase in matrix cracks. Blade holes are also a manifestation of crack expansion; Pan et al. [93] proposed a new method using sparse Bayesian learning beamforming to suppress ambient acoustic interference. Numerical simulation results showed that the inherent frequency of the acoustic emission signal tends to decrease in wind turbine blades in the presence of holes. To gain a deeper understanding of crack emergence, expansion, and interaction in the blade laminate, modal acoustic emission (MAE) and peak frequency methods were used to study the damage and evolution of four different layup composite laminates. Combining the frequency components and acoustic emission energy, the corresponding surface and internal ply cracking events were analyzed. Other factors affecting matrix cracking were also studied [94].

Wind turbine blade matrix cracks further evolve into delamination damage. Blade delamination is a very serious damage problem in glass/epoxy laminated composites, leading to a reduction in structural strength and stiffness. Saeedifar et al. [95] used cohesive zone modeling and acoustic emission techniques to understand and predict the onset and progression of delamination. Ihn et al. [96] analyzed parameter changes during the evolution of delamination damage by extracting the characteristic parameters associated with crack expansion. Fremmelev et al. [97] investigated the defect development of 52 m blades inflicted with different artificial damages by AE signal monitoring. This study explained the damage propagation around the sensor by measuring the increase in the number of AE hits. The AE sensors were also compared with other sensors, and it was found that due to the high attenuation of composite materials, the coverage area of AE was relatively small. Therefore, this may be a limitation of the application of AE sensors in large blades. The fatigue damage growth process caused by the blade damage source was also successfully detected by an acoustic emission sensor [98]. The AE signal was also found to correlate with delamination growth and channel cracking during fatigue loading. The experimental results finally confirmed that the further development of blade crack was stratified defect damage, and the stratified length increased to 0.3 m. In addition to the focus on the evolution of matrix cracks into delamination failure damage, the number and location of delamination instances on the interlaminar damage mechanism of the blade are also of interest. On the one hand, the AE technique was used to investigate the effect of blade delamination defects on interlaminar damage and acoustic emission response characteristics. Loading tests on composite specimens with two different delamination areas initially indicated that the delamination defects located in the middle of the shear surface had little effect on the cross-sectional load-carrying capacity. However, with an increase in the delamination defect area, the damage in the high-stress area on both sides of the deviated shear plane also increased. The AE relative energy, amplitude distribution, and other parameters were also reported to significantly change [99]. On the other hand, Zhou et al. [100] monitored the interlaminar shear characteristics of blade delamination in tensile tests and discussed the damage mechanisms. AE response results were obtained for four different instances of delamination specimen damage and evolutionary processes. Because blade delamination damage also affects blade material buckling and post-buckling activities, Sobhani et al. [101] monitored samples with three different damage types using AE techniques with Teflon films inserted at the interlaminar interface. Then, the influence of the number and location of delamination incidents on the evolutionary process of flexural damage in laminated composites was explored. Wind turbine composite blade delamination damage studies clearly indicate that threshold parameters need to be set when using AE parameters (number of impact hits, energy, and amplitude). However, their values are not uniform and depend on different research experimental settings or research objectives. The choice of threshold values may have an unavoidable effect on the accuracy of the experimental results. Therefore, a reasonable threshold value also needs to be carefully chosen in conjunction with specific experimental scenarios.

When layered damage occurs in wind power blades, it is usually accompanied by adhesive failure (fiber/matrix interface debonding, bonding joint debonding, etc.). Therefore, Zhou et al. [102] fabricated adhesive joints of two composite laminates with and without defects. The causes of failure of the adhesive joints were analyzed by monitoring parameters such as AE relative energy, amplitude distribution, cumulative hits, and duration, with two stages of damage evolution and failure. In addition, AE was used to monitor the damage mechanism of the cylinder-shell bonded joints of wind turbine composite blades with different overlap lengths under torsion experiments, as well as the causes of adhesive joint damage [103]. Zarouchas et al. [59] carried out tensile and compression experiments on single-lap bonded joints using the AE technique and determined the ultimate tensile, compressive, and elastic properties of adhesive specimens. The study also correlated the results of the AE activity frequency analysis with damage events. The results indicate that porous microcracking, macroscopic cracking, and fiber fracture are the three main mechanisms of blade damage. More importantly, macroscopic cracking is the main damage mechanism. Blade interfacial failure and hybrid failure were also observed in the experiments. Sun et al. [104] performed mixed-mode bending experiments on 53 adhesive specimens, which were set up with precracking with containing adhesions parallel to the interfacial fibers. A comparison between AE energy curves and AE hit count curves showed that the former indicated crack sprouting more clearly. Bond joint detachment also accelerates other structural failures, including buckling in the rear blade edge region and damage between the shear-resistant web and wing beam cap caused by detachment. Finally, Bak et al. [105] investigated the three main joint failure modes in composite materials: bonded, riveted, and hybrid joints. Not only were the AE counts of different damage types counted during monitoring in relation to the cumulative AE counts, but the main frequency ranges of different failure events were also obtained using FT processing.

The use of fiber-reinforced materials accounts for 30% of the whole blade manufacturing materials, in which the main blade beam, shell, and other structures are mainly used. Therefore, it is necessary to explore the evolution of fiber damage for leaf fabrication, maintenance, and repair. Ramirez-Jimenez et al. [106] investigated the main failure modes of fiber fracture, matrix cracking, and fiber debonding using homemade materials with different fiber orientations. The main frequencies were plotted on the power spectrum by FFT, and the obtained frequency clusters were defined. Zhou et al. [107] studied the damage process of composites with unidirectional or multidirectional fiber prebreak defects by the AE technique. When unidirectional fiber prefracture composites were loaded to about 30%, significant matrix and interface damage appeared at and near the defect location; when loaded to about 60%, significant interlaminar shear damage appeared. In contrast, when the multidirectional fiber prebreak composites were loaded to 60%, significant matrix damage appeared at the fiber prebreak. Mi et al. [108] conducted uniaxial tensile experiments on three types of GFRP composites to investigate the relationship between structural failure and prefiber weaving. The authors found that axial fiber fracture was closely related to oblique fibers, whereas shear forces caused by oblique fiber interweaving changed the amplitude, the number of bells, energy, and duration values in the AE signal.

Studies in the literature show that different types of blade damage formed by the same composite material can be clearly observed through the results of time–frequency transformation and that their frequencies are distributed in different types of intervals but that the one-to-one correspondence relationship is not affected. However, due to the complex composition of blade composite materials, the anisotropy characteristics are prominent, and the same type of damage frequency interval is a very different phenomenon. Therefore, individual uniqueness should be considered in the analysis of leaf damage types. Although frequency-domain analysis can be used to determine the type of damage, its results are not extensive. Therefore, research on other new key characteristic parameters may indicate the type of damage.

#### 2.2.2. Wind Power Blade Damage Source Localization

**Figure 5:**
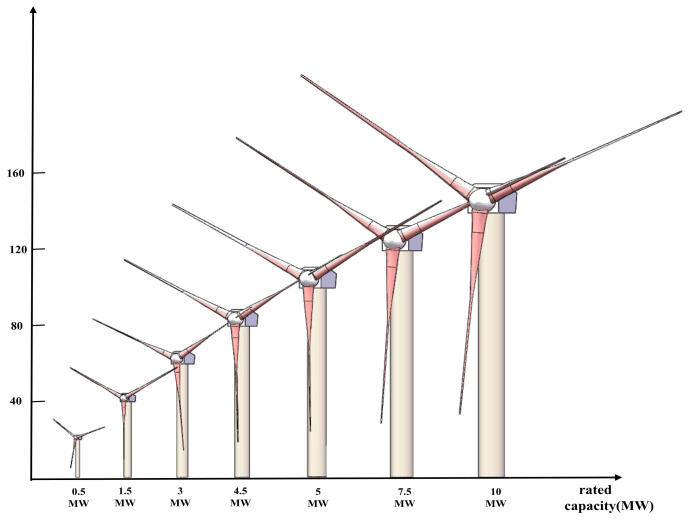
Development of large turbines over the years. Based on data retrieved from [72].

Wind power generation devices convert mechanical energy into electrical energy; the blade is the main structure that affects the power generation capacity. In order to improve the power generation efficiency, lighter and larger blades are used in large numbers [109], as shown in Figure 5. In recent years, larger blade diameters have been designed and applied to improve power generation efficiency. Accurate detection of the location of the damage source is crucial for accurate blade maintenance work.

**Figure 6:**
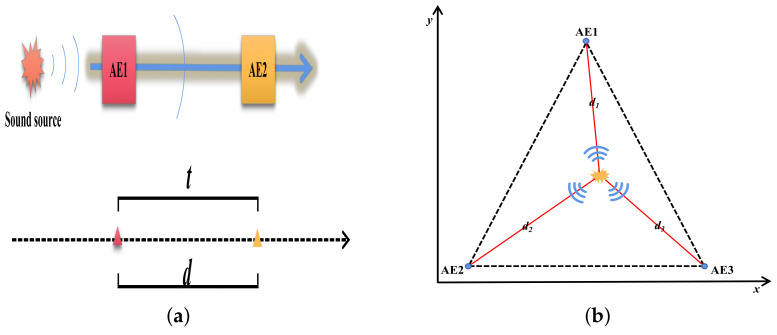
Acoustic emission source location. (**a**) Linear location of the AE source; (**b**) acoustic emission source plane location.

Analyzing the linear propagation velocity of the AE signal, as in Figure 6a, and calculating the AE signal wave velocity using AE1 and AE2 placed at a fixed distance for use in calculation of the damage source location is a commonly used method for locating the damage source. Such a method is commonly used to calculate the wave speed by setting up a pencil core break measurement experiment and calculating the arrival time difference and distance difference between the two sensors. Linear AE source localization was able to match the damage better only when the AE signal duration exceeded 200 us [100]. The accuracy of localization using the two-sensor method does not meet the requirements of the task. Therefore, Liu et al. [110] proposed a time arrival method based on four AE sensors placed in a square arrangement to locate the main defects within the blade damage area. The coordinates of the intersection point of two curves formed by two sensors, i.e., the AE source, were determined. To further improve the accuracy, triangulation was proposed and applied to damage source localization, as in Figure 6b. Gómez Muñoz et al. [111] proposed a new method for damage localization in composite materials using three AE sensors, which localizes the maximum error to less than 9 mm. However, the sensor’s fixed threshold setting may introduce delayed triggering, loss of low-amplitude information, or interference by undesired noise. Tang et al. [98] first verified a 45 dB monitoring threshold with only a 7.9% false alarm rate using a pencil core fracture attenuation test on the blade surface. To further improve the accuracy, the authors selected any three of the four sensors in the region of interest to form an approximate triangular region. Then, the system composed of four equations was solved to obtain the damage source coordinates, and the optimal number of sensors in the monitoring region was provided for calculation.

Ciampa et al. [112] proposed a new AE source localization algorithm based on the analysis of recorded signals from six sensors. To obtain the initial coordinates of the defect, the value of the squared mode of the continuous wavelet transform (CWT) was used to identify the time of arrival (TOA) of the bending Lamb wave (A0) mode and combined with a global search and a local Newton iteration method related to the backtracking method. When the localization error is less than 3 mm in anisotropic composites and less than 2 mm in sandwich panels, the experimental results imply that the study method does not require knowledge related to the anisotropic group velocity and material thickness of the waveform. However, the Lamb waves of composite materials excited by forces actually have two modes—a high-frequency signal (S0) and a low-frequency signal (A0)—which are influenced by the force of excitation and the angle of the plate surface. Baochun et al. [113] analyzed and verified the wave velocities of different modes of Lamb waves when the acting force was perpendicular and parallel to the structural plate and determined that the Lamb wave (S0) mode was generated by the damage. Furthermore, a new method was proposed to determine the impact damage based on the Lamb wave (S0) mode and to determine the location of the damage source using the time arrival difference of a specific intrinsic mode function in the EMD decomposition of the Lamb wave (S0) mode. Considering the effect of anisotropic characteristics on the location of the damage source of composites, Koabaz et al. [114] measured and calculated the envelopes corresponding to different fiber orientations to extract the energy velocities of the excited modes to calculate the guided mode velocities. The results show that even when the complex nature of different propagation directions of anisotropic composite plates is considered, the vibration source points can still be correctly predicted.

Linear or planar localization still uses the forced assumption of constant wave velocity in all directions for homogeneous materials, which can be limiting for anisotropic and non-homogeneous composites. Furthermore, anisotropy can lead to complex scattering phenomena, which complicate wave propagation. Moreover, geometrical features such as holes, curvature, and thickness variations can affect the signal propagation path. Zhang et al. [115] investigated the effects of multimodal effects and dispersion phenomena on wave velocity determination when AE waves propagate in a wind turbine blade thin-plate structure. Rumsey et al. [116] carried out fatigue damage testing on wind turbine blade composites; the results of their study show that acoustic emission waves are highly anisotropic and decay rapidly in energy during propagation. Sause et al. [117] studied the effect of test sources (such as pencil lead breakage and piezoelectric pulse generators) on the source sensor distance and analyzed the effect of dispersion and attenuation effects on the acoustic emission characteristics during the propagation of Lamb waves. Considering objective interference factors, a new precise positioning method was proposed. First, the time-frequency results processed by Hilbert–Huang transform were used for material anisotropy sound velocity compensation, and initial localization was performed using the four-point circular arc method. Then, the parameters of the probabilistic neural network were optimized using the chaos algorithm in the fish swarm algorithm and the improved Drosophila optimization algorithm to further improve the localization accuracy [118]. Based on the time-of-arrival method used to calculate the AE source location, the node recording start time or threshold selection can more or less affect the accuracy of the results. Kirikera et al. [119,120] proposed the use of a structural neural system (SNS) for damage detection and localization of a test blade with a length of 9 m. The most important feature of this system is the selection of the first-wave arrival time to localize the damage, avoiding the interference of wave propagation type.

**Figure 7:**
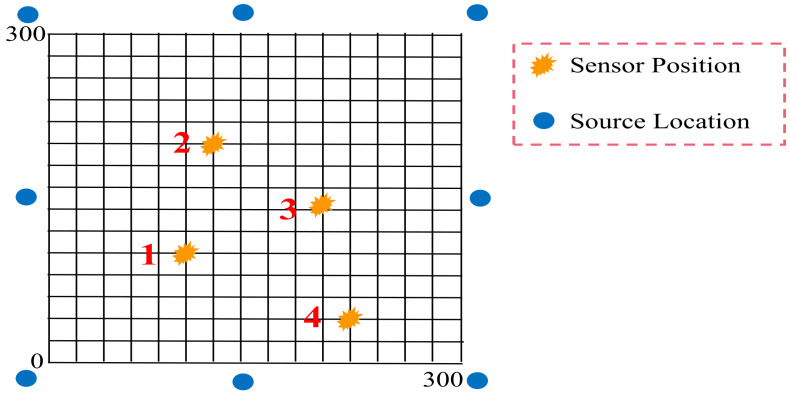
Layout of 300 mm “Delta T mapping” grid sensors.

A class of methods that can be outlined as a map comparison was applied to sound source localization. Eaton et al. [121] proposed a new method for AE source localization of composites applicable to anisotropy, namely Delta T mapping, as shown in Figure 7. By using each sensor to record the ΔT value of the real AE time and superimposing the constant line equivalent to the real event on the sensor recognition map to obtain a contour map, we can find the convergence point, namely the location of the sound source. This method significantly outperforms the traditional TOA localization method in terms of training time and localization accuracy. To further improve the localization accuracy, a new Delta T mapping and parametric correction technique (PCT)-based was proposed for damage source localization [122]. The results showed that the method improved the positional accuracy of matrix cracking and delamination identification. Han et al. [123,124] successively proposed a damage index-based database mapping source localization algorithm and an energy profile mapping-based localization algorithm. They found that the AE events coincided with the blade stress distribution and damage location. However, this method is affected by geometric variations at the bond edge, which is 1000–1500 mm from the blade root. Maillet et al. [125] proposed a new waveform-based procedure to select AE events generated by damage and automatically generate energy ratio versus position maps for damage event localization using the Akaike information criterion (AIC) technique for accurate localization.

Finally, recent studies in the literature have focused on AE signal acquisition methods and the ability of feature parameters to behave differently under different failure mechanisms. Bouzid et al. [126] used in situ wireless structural health monitoring (SHM) techniques combined with low sampling rates to perform localization of features extracted from mixed AE signals. The AE signals were subjected to relevant signal processing techniques to obtain parameters for different types of associated feature damage. However, it was stated whether parameter selection is the best characterization of the relevant study content. To obtain a clear understanding of the information contained in the feature parameters and their impact on the interpretation of damage accumulation, Xu et al. [127] used three algorithms to evaluate the dynamic characteristics of 15 conventional parameters recorded for the AE of bonded composite single-lap bonded joints with static tensile loading. The results of their study indicate that different AE features do exhibit different functionalities, including feature parameters applicable to the identification of different types of damage patterns, the best description of the damage process, and those in between.

### 2.3. The Advantages and Disadvantages of AE Signal-Based Wind Turbine Blade Defect Detection Technology

#### 2.3.1. Advantages of AE Detection

(a) AE structural integrity inspection can be performed without damaging the original structure of the material being inspected and is a reliable passive, nondestructive testing method. (b) AE inspection technology has real-time capability and is highly sensitive to any acoustic process or mechanism. (c) AE technology can detect damage earlier than vibration inspection technology in structural integrity inspection and has the advantage of early warning. (d) AE technology can locate the damage area while detecting structural integrity.

#### 2.3.2. Disadvantages of AE Detection

(a) AE signals are usually released in the form of acoustic waves when structural damage such as cracks occurs within the material, so not all types of damage produce strong detectable signals. (b) Many extraneous external noise sources can cause interference at the actual AE inspection site. (c) The anisotropic nature of the composite material itself causes the AE signal to decay rapidly in energy as it propagates, requiring a large number of sensors and an inconvenient installation procedure. (d) AE inspection systems require fast real-time data collection, a large amount of data, and, therefore, high inspection costs. (e) AE technology relies on material surface wave detection; high sensitivity and a high acquisition rate of AE sensors are required.

## 3. Wind Turbine Blade Defect Detection Based on Aerodynamic Noise Signal Analysis

### 3.1. Introduction of Wind Power Blade Aerodynamic Noise

**Figure 8:**
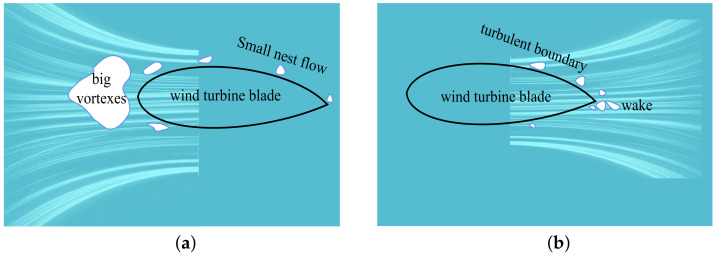
Main aerodynamic noise types of wind turbine blades. (**a**) Turbulent noise; (**b**) turbulent boundary layer noise.

Wind power noise is divided into mechanical noise and aerodynamic noise. Mechanical noise is generated by the internal parts of the wind turbine nacelle (such as high-speed gearboxes, generators, and yaws). On the other hand, aerodynamic noise can be explained as being generated by the interaction of the cyclically rotating blades with the air blowing towards them. Aerodynamic noise is the main noise source of wind power and can be classified into three types: low-frequency noise (periodic blade rotation noise), turbulence noise (Figure 8a,b), and trailing edge noise (aerodynamic noise). Among them, low-frequency noise is usually independent of blade surface damage. The blade surface characteristics and trailing edge shape are the key factors affecting inflow turbulence noise and trailing edge noise changes [128].

**Figure 9:**
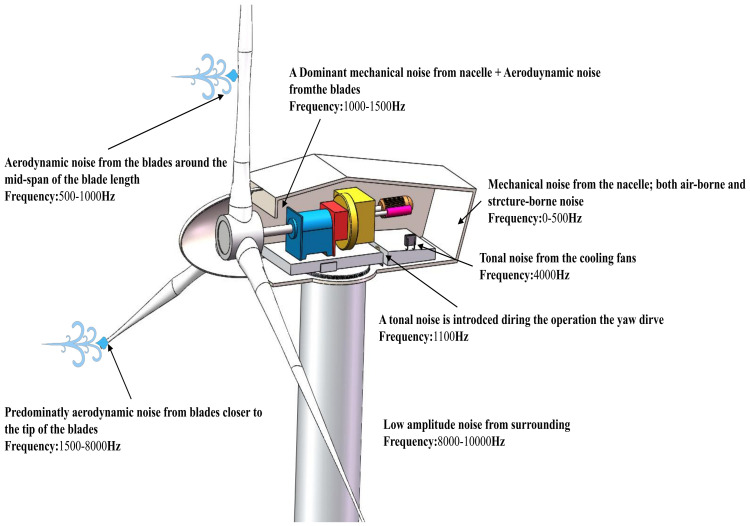
Noise frequency distribution of a wind turbine. Based on data retrieved from [133].

Wind power blade trailing edge cracking (TEC) is generally an early blade health problem. Zhang et al. [129] proposed a prediction method combining computational fluid dynamics (CFD) simulations and semiempirical models. An airfoil cross-sectional analysis of the blade trailing edge with and without a 3 mm gap crack defect successfully explained that blade trailing edge cracking is the cause of the accompanying acoustically sharp “whistling sound”. Genescà et al. [130] analyzed the differences between the noise signals of rotor blades with three different surface treatments using a linear microphone array. They found that the blade surface shedding sound pressure level was the highest. To determine whether the material had been damaged, Jüngert [131] used microphones to record the sound of repeated excitation of the blade by a hammer and compared it before and after. Subsequently, to avoid errors due to technical changes in the tapping operation by technicians, a new method was proposed using instrumented percussion equipment to generate a fixed, controllable signal combined with a local acoustic resonance spectrum [132]. In addition, Ramachandran et al. [133] studied wind power mechanical noise and aerodynamic noise using an advanced deconvolution-based beamforming algorithm. As shown in Figure 9, the frequency distribution ranges of both noises were observed, and the noise of the blades was determined to be mainly influenced by trailing edge noise.

### 3.2. Wind Turbine Blade Damage Detection Based on Beamforming or Acoustic Transmission Parameter Variation

Beamforming is a method of processing data received from a microphone array to produce a visual image representing the radiation pattern of the sound source and the relative intensity of the sound source. Usually, microphones can map the received array to a plane after processing to show the location of the sound source distribution. Microphone arrays used to locate motion sources can effectively distinguish aerodynamic noise distributed at different locations of wind turbine blades and are promising for wind turbine blade damage detection [134,135,136,137]. Aizawa and Poozesh et al. [138,139] investigated phased array beam formation and two beam formation algorithms (CLEAN and CLPSR) for wind turbine blade damage detection. The experimental results show that the CLEAN-based algorithm can identify the maximum source. In contrast, CLPSR is more effective in locating sources generated by damage alone. The CLPSR algorithm also has the potential to evaluate the fault size based on the sound pressure level (SPL) of the fault emission.

**Figure 10:**
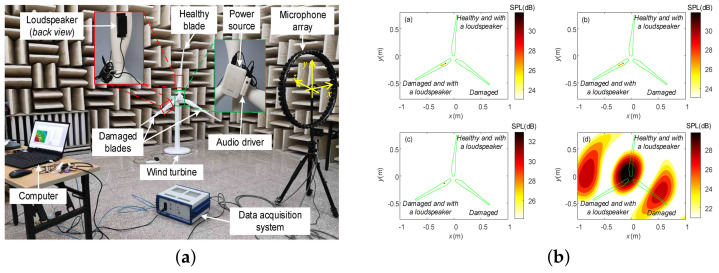
Beamforming algorithm system and results. (**a**) Experimental setup for blade damage identification; (**b**) acoustic maps of healthy blades and blades with cracks [142].

The development of beamforming in wind turbine blade damage detection is subject to two key limitations. On the one hand, conventional beamforming wind power, although available for blade damage detection, is limited by spatial resolution, severe side flap contamination, and computational timeliness. In order to improve the spatial resolution and accuracy, Sun et al. [140,141] proposed a new algorithm for sparsity-based acoustic field reconstruction and regularized the acoustic problem using the generalized minimax-concave (GMC) penalty function. In addition, the experiment investigates the performance capability of different acoustic parameters on blade identification under specific measurement conditions. The results show that a reasonable matching of key parameters or measurement conditions can improve recognition accuracy and provide a method to determine the parameters. Finally, the experiments also revealed the potential influence of damage on the acoustic field. Another adaptive compressive beamforming method based on a generalized minimal–maximum concave penalty function was used to reconstruct the sound to identify blade damage [142]. Figure 10 shows the composition of the beamforming hardware acquisition system and the results of the beamforming algorithm for blade damage identification. On the other hand, although increasing the number of channels improves the accuracy of beamforming detection, it also increases the cost. A sparse sensor array optimization method for composite wind turbine blades was proposed, and an improved redundant second-generation wavelet transform (IRSGWT) algorithm based on domain coefficients was used to denoise weak signals and validated with simulated damage on wind turbine blade laminates [143].

In addition, considering that only monitoring the structural state of the blade cannot meet the demand in practical applications, a reliable early warning mechanism is also needed to satisfy the optimal maintenance time of the blade. Li. [144] designed and developed a nondestructive detection system based on acoustic signal fusion using the minimum variance distortion-free response (MVDR) beamforming technique to enhance weak signals and suppress interfering signals and introduced two early warning measurements to improve the early warning robustness of the system.

**Figure 11:**
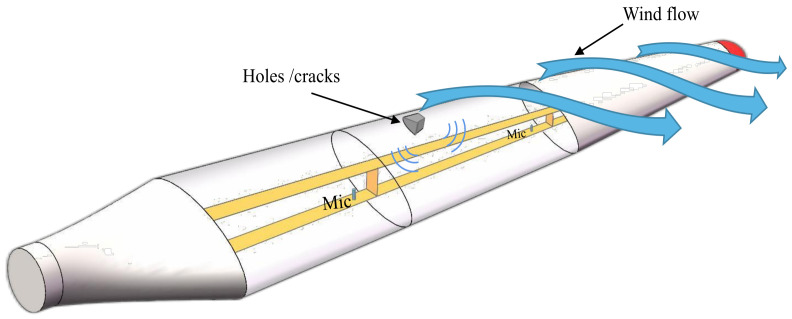
Passive detection method based on aerodynamic noise.

Acoustic transmission loss variation is a typical manifestation of wind turbine blade damage. The aim of the method based on acoustic transmission parameter variation is to study and measure the acoustic parameter variation in the external and internal air domains of the wind turbine blade cavity, as shown in Figure 11. Based on the acoustic transmission characteristics of the noise generated by acoustic excitation when air flowing through the blade enters the cavity structure, Beale et al. [145] investigated the influence of different damage locations and damage degrees on the detection performance of the blade section in a wind tunnel. The effects of various damage locations and damage levels on the blade cross section in the wind tunnel on the detection performance were investigated. The authors confirmed that the proposed method can detect hole-type damage with a diameter of 0.32 cm and crack damage with a length of 1.27 cm.

**Figure 12:**
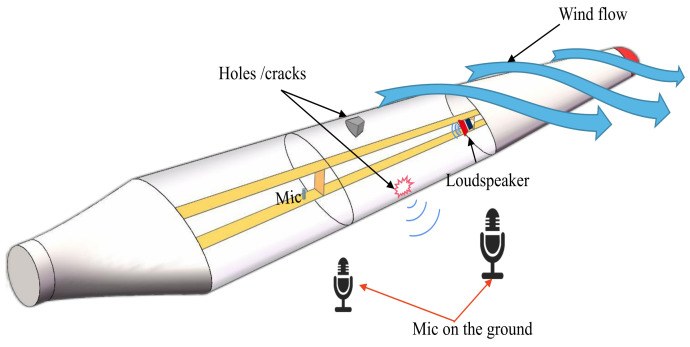
Active and passive detection method based on aerodynamic noise.

Traylor et al. [146] proposed a generic computational method capable of predicting the sound pressure distribution within the blade cavity affected by noise generated by high-frequency flow and conducted an example study on a 5 MW wind turbine blade model. The results show that the proposed method can successfully detect damage in the front half of the blade cavity at the root position of the wind turbine blade. Moreover, the signal frequency variation can not only indicate the type of damage that has occurred but also possibly be used for location determination. An active damage detection method relying on the detectability of sound propagation changes at the boundary of the structural cavity was studied for the measured sound pressure response outside the cavity of wind turbine blades. Experiments were also conducted to explore the effects of the blade damage level, microphone placement, and other conditions on the detection performance. This study proves the feasibility of the active acoustic damage detection method in cavity structural integrity and full-size wind turbine blade damage identification [147]. Inalpolat et al. [148] proposed a damage detection method combining active and passive approaches by taking advantage of the property that blade structural deformation or damage can cause loss in the transmission process of blade sound into or out of the cavity. Four progressively complex experiments were conducted, investigating the combined cavity, blade section, field turbine blade, and turbine fatigue damage. The results show that active–passive detection is feasible for identification of blade crack or hole-type damage. The active–passive detection process is shown in Figure 12; both microphones and loudspeakers are installed in the wind turbine blade cavity, and the loudspeakers can provide sound sources during active detection. The low number of pneumatic noise sensors required for this method is one of the advantages of noise-based detection wind turbine blade technology, and the technique of using wireless sensing methods to detect surface damage to blades was investigated [149]. Moreover, Barber et al. [150] proposed a cost-effective MEMS-based acoustic wireless measurement system.

To optimize the acoustic-based wind turbine blade health inspection system and guide the inspection method, the effects of damage location, damage size, sound source location, and microphone location on the detection rate using structural–acoustic coupling were investigated in [151]. Traylor et al. [152] proposed a blade aerodynamic reduced-order acoustic–structural coupling modeling approach for the study and analysis of five damage locations and four damage sizes at specific acoustic frequencies (1, 5, and 10 kHz) with respect to a healthy baseline. The results show that the SPL increased by more than 3 dB in 22 of 36 anterior cavity damage cases, whereas only 6 of 24 cases in the posterior cavity increased by more than 3 dB. On the other hand, the experiments also revealed SPL, damage location, damage size, and frequency as key factors affecting the success of identification.

### 3.3. Wind Turbine Blade Damage Detection Based on Other Acoustic Methods

Changes in the acoustic cavity response function of wind turbine blades are also a form of damage manifestation. Two damage detection methods using passive reconstruction of the impulse response function or Green’s function of the existing noise mechanism in the structure are used for blade damage studies and have been demonstrated in marine acoustics. Tippmann [153] studied the reconstruction of forward and backward time-domain Green’s functions between any two detection points on a structure using the derivatives of the mean of the coefficients of the mutual correlation function for blade damage detection. In addition, the diffusion field of the aerodynamic noise around the blades of an operating wind turbine can be calculated by reconstructing different types of Green functions [154,155] to detect damage to wind turbine blades. Another sparse array imaging technique (matched field processing) based on acoustic fields providing unique information on structural modal wave propagation was used to reconstruct impulse response functions in ambient noise to detect blade structural damage [156]. Detection techniques combining Green’s function and the impulse response function have also been investigated for blade damage detection and spatial localization to identify structural sources [157,158].

### 3.4. The Advantages and Disadvantages of Wind Turbine Blade Defect Detection Technology Based on Aerodynamic Noise Signals

#### 3.4.1. Advantages of Aerodynamic Noise Detection

(a) It is based on blade aerodynamic noise damage detection, without unit outage, non-contact, and continuous detection; (b) Aerodynamic noise is easy to collect and difficult to mask; (c) Aerodynamic noise acquisition methods are abundant, sensor placement is simple, and detection cost is low; (d) Aerodynamic noise has the potential to detect structural integrity and the location of blade damage and can successfully detect aperture damage as small as 0.32 cm in diameter (even holes as small as 0.16 cm in diameter combined with data-driven algorithms) and crack damage as long as 1.27 cm; (e) Due to different damage types of blades, the natural frequency of radiated noise changes and has the potential ability to identify damage types.

#### 3.4.2. Disadvantages of Aerodynamic Noise Detection

(a) Blade aerodynamic noise contains a large number of frequency components with a wide frequency distribution range. The main frequency distribution has no obvious rule, and the distribution is random; (b) Wind power blades work under adverse conditions. When the wind speed is too high, the background noise is increased, and interference is obvious; (c) No unified standard specification has been established for the installation position of acoustic acquisition sensors in the process of blade aerodynamic noise acquisition; (d) The developmental process of leaf damage is uncertain, and the relevant acoustic signal damage characteristics are not fully understood; (e) Due to a lack of aerodynamic noise data, data-driven combination is relatively difficult.

## 4. Machine Learning Combined with Acoustic Signals for Wind Turbine Blade Damage Detection

### 4.1. Machine Learning

The concept of machine learning was first introduced in 1959 [159]. The concept has taken 60 years to develop and is widely used in various industries, such as computer vision, natural language processing, and biometric recognition. Machine learning also covers the field of deep learning that is being studied today. Machine learning methods can usually be divided into two categories: supervised learning and unsupervised learning. No matter which type of method, feature extraction and model selection have a decisive impact on the final recognition effect. Key feature extraction can determine the upper limit of the recognition effect, whereas selection of the appropriate model enables the result to approach the upper limit. Past case studies have demonstrated the effectiveness of machine learning algorithms in solving difficult problems in the area of damage identification, as well as the potential for fault detection in some parts of wind turbines [160,161].

### 4.2. Wind Power Blade Damage Identification Based on AE Signal Combined with Machine Learning Algorithm

In Section 2 of this paper, we reviewed the traditional AE signal analysis methods, which are promising technologies for health monitoring of composite structures. However, traditional analysis methods are difficult to automatically interpret and distinguish AE data failure modes. Solving AE data fault classification and identification is actually also a pattern recognition problem. Therefore, the combination of AE signals with appropriate machine learning algorithms can be an appropriate solution to the damage identification problem. In [162], the authors applied “NOESIS” pattern recognition and neural network software to perform unsupervised analysis of AE data. The separation of categories, which is not possible with traditional AE analysis methods, was successfully achieved. In the following section, we present common algorithms for wind turbine blade health monitoring that combine machine learning with AE signals.

#### 4.2.1. Clustering Algorithms

Clustering algorithms can be summarized as dividing similar data into the same group while maintaining distinct differences between groups. Pattern recognition can be defined as the discovery of potential patterns in data to aid in classification and a better understanding of data distribution patterns. Liu et al. [110] used a one-round cross-validation approach for dataset partitioning of AE data and used a bipartite k-means clustering approach in combination with defect localization information to complete the damage analysis. Nair et al. [163] used the complete link algorithm and the PCA algorithm to select a data feature set in combination with the k-means algorithm to correlate the data results with the associated fault damage. Zhang et al. [164] proposed a feature selection method by calculating the temporal signal density of damage patterns for feature selection and using the k-means algorithm to identify damage patterns in tensile experiments on glass fiber composites containing delamination defects. Tang, Tang, et al. [165] collected 21-day fatigue load AE data of a 45.7 m blade and used the k-means algorithm to identify AE activity for different fracture mechanisms and found four modes of fiber failure: matrix cracking, fiber matrix debonding, and delamination (interlaminar failure). They also evaluated the influence of k-value selection on damage clustering results using the silhouette index and the Calinski–Harabasz index and found that k = 4 was the optimal value. K-means clustering is an unsupervised algorithm used to determine the uncertain origin of unknown events. Therefore, the k value and initial clustering center should be determined first when using this method. The choice of k value has a considerable influence on the running time and results of the whole model. The fuzzy c-means (FCM) clustering algorithm was proposed later than the k-means algorithm. The FCM algorithm introduces “fuzziness” treatment to the model, which is an improvement of the k-means clustering algorithm. Marec [166] extracted AE signal features using CWT and discrete wavelet transform (DWT) algorithms and studied typical damage AE signals, such as matrix cracking and fiber damage, using a combination of PCA and FCM unsupervised clustering model algorithms. Azadi et al. [62] used wavelet packet transform (WPT) and FCM to study failure mechanisms such as matrix cracking, fiber fracture, and fiber detachment from the matrix of composite materials under different tensile loading rates and found that the method was effective in identifying the debonding failure mechanisms. Mohammadi et al. [167] used AE in combination with wavelet and FCM clustering methods to identify the damage mechanisms of standard open-hole tensile (OHT) laminates, and their single-step processing algorithm was also compared with finite-element calculation methods, proving the effectiveness of the method.

In recent years, many new clustering algorithms have been combined with AE signals for wind turbine blade damage identification. Ramasso et al. [168] proposed a cluster fusion pattern recognition algorithm that automatically selects multiple feature subsets using damage sequence entropy, estimates the optimal number of clusters to represent the AE data stream structure, and provides the cumulative load threshold interval required to activate specific damage. Experimental results show that the algorithm is able to cope with damage sensitivity while capturing damage dynamics and onset points. Xu et al. proposed fast search and find of density peaks (CFSFDP) to achieve damage pattern identification for interface debonding, matrix cracking, and fiber fracture and to determine other reasonable acoustic emission features by combining Laplace scoring with the correlation coefficient evaluation index. Subsequently, they explored a pattern recognition method without signal denoising, the CFSFDP method of WPD, and applied it to the pattern recognition and outlier detection of 59.5 m long composite wind turbine blades. A clustering algorithm based on Shannon entropy combined with WPD was also proposed and validated on non-aging and hygrothermal aging specimen damage patterns [169,170,171]. Compared with CFSFDP clustering, k-means clustering results change significantly with changes in the number of clusters when selecting clusters, which may lead to differences in clustering results between the two algorithms. Ech-Chouda et al. [172] developed the incremental clustering (IC) algorithm for identifying and analyzing the types of unidirectional ply GFRP damage mechanisms. IC has more advantages than k-means methods in classifying AE signals such as matrix cracking, fiber breakage, and delamination and in building a learning database. AE clustering based on k-means classification shows the existence of four fully separable clusters without any overlap. The IC algorithm clustering algorithm is not only suitable for four separable clusters, but the overlap between clusters also shows the physical properties and states of AE signals.

The combination of the above AE signals with clustering shows that AE signals of leaf damage can be divided into four distinct classes, namely matrix cracking, fiber matrix degluing, fiber fracture, and delamination.

#### 4.2.2. Classification Algorithms

A classification algorithm can be generalized as a guided learning process to make predictions for a single individual in a known total number of categories. Zhang and Jia et al. [173,174] used BP neural network models to study leaf defect damage identification, the former using the combination of variational modular decomposition (VMD) and energy entropy to construct feature vectors and the latter using feature clustering with dimensionality reduction processing. Experimental results show that both methods can achieve 90% accuracy in leaf damage identification. Wirtz et al. [175] proposed a pattern recognition method based on STFT feature extraction and support vector machine (SVM) classification, using probabilistic estimation to evaluate the reliability of SVM models under damage evolution and variable loading conditions and analyzing the temporal data compression rate of STFT, as well as the tradeoff limit between time and frequency resolution. Jiang et al. [176] proposed a wind turbine blade damage diagnosis method based on a combination of complete noise-assisted total empirical modal decomposition (CEEMDAN) and SVM, which achieved 96.7% accuracy in blade defect identification. Loutas et al. [177] proposed an innovative data-driven framework, the non-homogeneous hidden semi-Markov model (NHHSMM), with multistate degradation and the RULE function to reduce fluctuation. They found that the confidence intervals of the model approach zero over time with an effect of increasing data.

**Figure 13:**
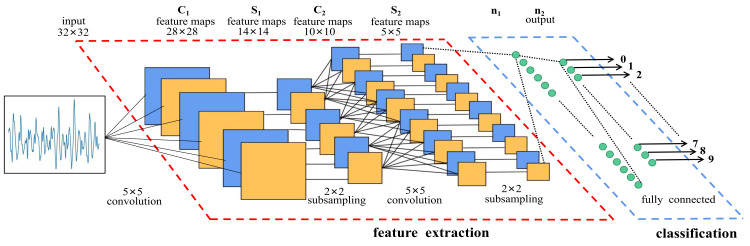
Convolutional neural network structure.

The deep learning model (Figure 13) has excellent automatic mining capability in image training, due to which the AE signal waveform can also be used as a training sample. Guo et al. [178] proposed a deep learning method for detection of fiber breakage, matrix cracking, and delamination, namely the InceptionTime model. The model contains five inception modules to reduce overfitting of small datasets and filtering of different lengths. In addition, parallel MaxPooling is used to stabilize the model to avoid small interference. The model achieves 99% correct classification with high accuracy on both raw AE time series and frequency-domain series data training and 100% correct classification in matrix cracking mode. The advantages of this research method include not only excellent classification performance but also the ability to solve the AE data imbalance problem. Barile et al. [179] trained image-based AE waveform classification using a convolutional neural network (CNN) with four different modes of AE waveforms obtained from Mel-scale spectral representation as the training and test sets. The model comprises nine hidden layers and uses ReLu activation functions instead of sigmoid and tanh functions, which were commonly used in the past. The results show that the overall prediction accuracy of the model can reach 97.9%, and the prediction accuracy of fiber fracture and stratified damage can reach 100%. However, many existing algorithms may be deceived by indirectly propagated AE modulated by reflected boundaries within the structure. Haile et al. [180] converted time series data into spectral time feature images and input the CNN model constructed by six hidden layers to perform the classification task. In addition, the sensitivity of the model to image occlusion was studied, and the generated heat map further confirms the importance of the initial pulse as the distinguishing feature of the direct propagation waveform. Finally, the training results of time series data prove that a CNN is superior to long short-term memory (LSTM) in terms of performance. Sikdar et al. [181] proposed a deep learning framework for neural networks combined with image enhancement techniques to generate training datasets, process AE signals using CWT, and extract discrete damage features from scale map images. Wind turbine blade damage accumulation can affect the distortion of the AE signal in the time or frequency domain waveform. M. Kharrat et al. [182] proposed an improved algorithm to guide the evolution of damage features and showed that both time and frequency domains distorted with damage accumulation, which, in turn, led to important changes in the AE features used in data classification.

Past studies on AE signals combined with data-driven algorithms have shown that the results obtained by deep learning algorithms are usually superior to the detection results of machine learning methods. The possible reason for this difference is that deep learning algorithms can learn advanced features from data. However, deep learning requires a large number of training samples, which may be one of the key difficulties limiting the practical application of deep learning in leaf damage detection. In the long run, acoustic emission signals combined with deep learning algorithms are expected to become one of the main technologies used to solve the problem of automatic blade damage recognition.

### 4.3. Wind Turbine Blade Damage Identification Based on Aerodynamic Noise Combined with a Machine Learning Algorithm

**Figure 14:**
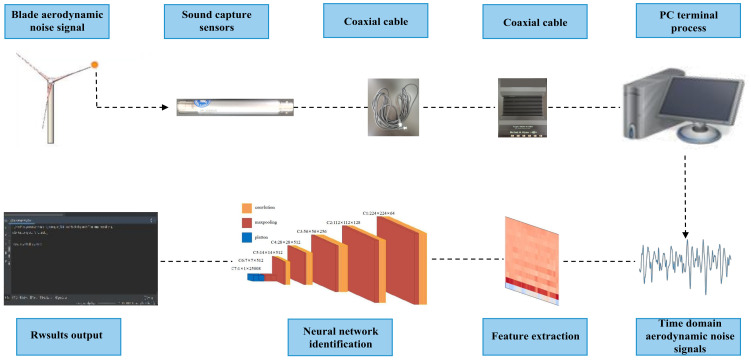
Machine learning diagnosis of blade damage based on aerodynamic noise signal combination.

The aerodynamic noise signal radiated by wind turbine blades carries blade mass health information. The related methods based on blade aerodynamic noise signal damage detection were reviewed in Section 3 of this paper. Although the traditional methods can detect damage, damage refinement and automatic classification are difficult. For example, beamforming algorithms can detect damage based on aerodynamic noise radiation at different locations, but it is difficult to determine the damage type or estimate the damage size. However, using appropriate data processing techniques makes it possible to classify and identify damage with different properties. In the following section, we introduce research on commonly used aerodynamic noise signal processing techniques combined with machine learning for wind turbine blade damage detection, as shown in Figure 14, which shows the whole process of wind turbine blade aerodynamic noise acquisition, preprocessing processing, fault feature extraction, and damage identification combined with a machine learning algorithm.

#### 4.3.1. Aerodynamic Noise Feature Extraction

Feature extraction is a data preprocessing process, as the original signal contains a large amount of redundant information that may not actually be relevant to the recognition task. Therefore, feature extraction is crucial for machine learning, and choosing an appropriate feature extraction processing algorithm can improve the upper limit capability of the model. In this section, we introduce the commonly used feature extraction algorithms and common machine learning models for wind turbine blade aerodynamic noise signals.

After damage occurs on the blade surface, the aerodynamic audio signal generated by the blade rotation process in air differs from the normal blade sound signal and is related to the type of blade damage. Solimine et al. [183] used a microphone placed inside the blade to capture the trend, variation, and peak of the blade cavity sound pressure; extracted the short-time energy, excess zero rate, and linearly predicted cepstrum coefficients; and input them into a training model after reducing the feature space dimension with low correlation using the PCA technique. Han et al. [52] first used a 1/6 octave to carve out the noise signal differences, combined with PCA to remove redundant information of energy features and feature dimensionality reduction to obtain key damage features; the accuracy of experimental classification results reached more than 98%. Hu et al. [184] analyzed the blade abnormal state using the collected wind power audio data, used FFT as a filtering tool for sound data, and obtained 264 key features from 1365 features in the time and frequency domains using a multiclassification logistic regression model. In addition, the authors explored the “whistling” sound and analyzed the possible causes. Kuo et al. [185] investigated the extraction of audio features by reconstructing audio files from eight different frequency ranges using DWT and employing missing value replacement. The damage detection method proposed in this experiment not only achieves better results in wind turbine blade damage detection but also has better generalization capability. When the sensor collects the blade aerodynamic noise, other noises in the environment are mixed with the blade noise to be collected; therefore, in order to improve the signal-to-noise ratio of the sound signal related to the blade damage, these noises that are mixed and collected unrelated to the blade damage should be screened and removed to the greatest extent possible. Peng et al. [186] analyzed the frequency band range and aerodynamic noise characteristics of both ambient noise and aerodynamic noise and proposed a noise reduction method and feature extraction method for wind turbine blade sound signals based on improved EMD and composite mean squared error. Compared with the traditional noise reduction method (EMD + relative entropy denoising method), the newly proposed method is widely applicable to different types of noise. The new method is widely applicable to sound denoising with different signal-to-noise ratios.

Mel-scale frequency cepstral coefficients (MFCCs) represent the most commonly used method for speech signal processing in recent decades. By proposing MFCC features for sound signals, Liu et al. [187] achieved 94.8% recognition accuracy; the field verification experimental results were also consistent with the wind turbine blade variation law. Zhang and Jiang [188] proposed a method to collect sound signals using sensors installed below wind towers and extract MFCC features from the noise-reduced data after high-pass filtering. The experimental results show that the 1, 2, 4, 6, and 8 dimensions of the 12-dimensional MFCC features were more obvious in distinguishing the faulty samples from the normal ones. Solimine and Inalpolat [189] set up simulated damage in the form of penetrating holes and cracks at three different locations (the leading edge of the front cavity, the side of the front cavity, and the side of the back cavity) and compared the MFCC, GTCC, LFCC40, and LFCC80 feature sets, finding that the LFCC40 and LFCC80 feature sets provided the highest detection accuracy in both hole-type and crack-damage-type damage and significantly outperformed the common MFCC feature set. If a damage location is given, a hole-type damage of 0.16 cm can be detected 100% of the time, and the accuracy of detecting cracks with a minimum length of 1.27 cm can reach 97%. The literature referenced above shows that the same signal processing method may achieve different results due to the location of the acquisition sensor or the radiated noise at different damage locations on the blade. Therefore, it is worth exploring the processing methods for aerodynamic noise of blades with high generalization ability. In addition, a low-frequency noise extraction method based on the spline envelope method and improved local mean decomposition was proposed to address the fact that the main components of blade aerodynamic noise are easily distorted in the modulation process [190]. The method was also successfully validated in real noise fields.

The radiated aerodynamic noise of wind power blades contains information about blade health state, but other environmental noise signals are inevitably saved when the blade aerodynamic noise is collected. Uncertain factors such as the location of the acquisition sensor, the location of blade damage, and the type of blade damage may affect the data quality. Therefore, the collected aerodynamic noise signals have a random frequency distribution and complex components and are difficult to analyze, resulting in differences in the performance of analysis results.

#### 4.3.2. Blade Damage Recognition Model

Zou et al. [191] used the k-means algorithm to separate a speech spectrogram into two classes when obtaining wind turbine blade aerodynamic noise data, separating the blade sweeping audio and quiet audio and inputting the sample data into the model for training after performing 0 and 1 sample sequence labeling. Dong [192] proposed the use of the DBSCAN density-based clustering algorithm, which not only does not require specification of the number of clusters for clustering but also allows for discovery of arbitrary shape clusters. The study was conducted by constructing a posterior number of sound signal features to input into the training model, obtaining a recognition accuracy of 94%. The method can discover the noise points in the signal, which is beneficial to the noise-containing frequency filtering process.

Regan et al. [193] investigated wind turbine blade damage (holes and edge cracks) by collecting data from laboratory-scale wind power models and using linear regression and SVM. The best characteristics of blade defect identification performance under multiple-excitation or single-excitation conditions were also discussed in the study. The results showed that the accuracy of wind turbine blade damage identification exceeded 98%. Ciaburro et al. [194] used the Boruta algorithm to select the most sensitive predictor of damage and trained it using the SVM model. The results showed that the accuracy of the SVM model could reach 91.8%. Liu et al. [195] proposed a new ITD-PCA-SVM classifier with a higher recognition rate and demonstrated a significant difference in the frequency bands of the damage signal. Chen et al. proposed an improved incremental bounded SVDD (IBSVDD) model for blade damage identification and found that the model performed best in training and improved prediction accuracy compared to other models (ISVDD and NISVDD). Recently, blade drainage hole health status has received attention. The newly proposed AR-ISVM model can retain most of the information through only a small part of the original sample. The method not only has a shorter training time than other incremental SVM classifiers but also has higher accuracy and F1 values than other classifiers (e.g., random forest, k-nearest neighbor, and XGBoost models) [196,197].

An acoustic monitoring model that combines DWT extracted features with a deep neural network (DNN) was proposed and applied to blade damage research. The method not only achieves satisfactory results in wind turbine blade damage detection but also has a positive generalization capability. For example, four different types of machine data were tested for wind turbines, pumps, sliders, and valves [185]. Yang et al. [198] used an Adaboost model to monitor the stratified regions of wind turbine blades in combination with a random forest model to classify the collected data. The model was experimentally compared in defect detection and region classification and achieved 88.57% accuracy in stratification identification and 78.33% accuracy in region classification. Tsai and Wang [199] proposed a novel hybrid CNN model for blade surface damage detection based on acoustic signals, which is a hybrid model introducing a masking module and a residual classifier with the functions of suppressing redundant information and quantifying the differences between masked and standard acoustic spectrograms. The model achieved excellent performance in terms of accuracy, precision, recall, and F1 score in the test set.

## 5. Summary and Prospects

Long-term wind power blade operation in harsh field environments using an efficient and convenient structural integrity monitoring method can provide a scientific reference for maintenance work timing. On the one hand, the working life of wind turbine blades can be extended, in addition to saving on maintenance costs. On the other hand, if the blade is damaged, maintenance is not timely, which can lead to greater disaster occurrence. In this paper, we reviewed existing technology used for wind turbine blade health monitoring, mainly focusing on the progress of wind turbine blade health monitoring technology based on acoustic signals (acoustic emission signals and aerodynamic noise signals) in recent years. We reviewed research on traditional methods of structural health monitoring based on acoustic signals in damage identification and damage location determination of wind turbine blades. The existing literature on the automatic identification and classification of blade damage based on acoustic signals combined with machine learning algorithms was also investigated, and the research progress was analyzed in depth. Possible directions for future wind turbine blade failure event detection technology are discussed below.

With wind power generation gradually trending towards more remote fields and deep-sea applications, blade size is rapidly increasing. The demand for remote, nondestructive, real-time, comprehensive, and accurate wind turbine blade health inspection is the main development trend of future wind turbine blade damage detection technology. This paper can serve as a reference for understanding damage detection based on acoustic emission signals or aerodynamic noise, as well as damage source location determination, which is important to further promote practical research and grasp the research development trend in this area.

## Data Availability

Not applicable.
